# La Grippe

**Published:** 1890-02

**Authors:** 


LA GRIPPE.—Sir Oscar Jennings, the noted English physician, states that La Grippe is a "bastard, pulmonary rheumatism." From this it would appear that the use of liq. tong. sal. (Tongaline) is particularly indicated for the relief of that trouble, which has proved such an epidemic in Europe and promises to do so in this country. In liq. tong. sal. we have tonga, anodyne; cimicifuga, anti-rheumatic, anti-spasmodic; sodium salycilate, anti-germinative; pilocarpin, diaphoretic; colchicon, anti-rheumatic, purgative, diuretic. It will be observed, therefore, that the action of Tongaline, which is exactly adapted for the indefinite kinds of rheumatism, should kill the microbe, and carry such out of the system through the natural channels. In some instances the use of quinine, antipyrin, acetanilid, aconite, benzoate of lithia, iodide of potassium, etc., may also be used in connection with liq. tong. sal. when indicated by the peculiar conditions of the case.

